# Recreational Nitrous Oxide-Induced Subacute Combined Degeneration

**DOI:** 10.7759/cureus.23409

**Published:** 2022-03-22

**Authors:** Yetunde B Omotosho, Grace W Ying, Richard Orji, Heeren Patel

**Affiliations:** 1 Internal Medicine, The Chicago Medical School Internal Medicine Residency Program at Northwestern Mchenry Hospital, McHenry, USA; 2 Internal Medicine, Chicago Medical School Internal Medicine Residency Program at Northwestern Mchenry Hospital, McHenry, USA; 3 Neurology, Northwestern Medicine, Regional Medical Group, McHenry, USA

**Keywords:** methylmalonic acid, subacute combined degeneration of the spinal cord, rare cause of vitamin b12 deficiency, nitrous oxide abuse, nitrous oxide myelopathy

## Abstract

Subacute combined degeneration (SCD) is myelopathy caused by vitamin B12 deficiency, leading to demyelination of the dorsal column located in the posterior spinal cord. Despite the high prevalence of recreational nitrous oxide use, its detrimental effects, including significant nerve dysfunction, are insufficiently recognized. We present the case of a 32-year-old male who was brought to the emergency department with complaints of paresthesia of his four extremities and lower extremity weakness. He was found to have B12 deficiency from chronic nitrous oxide abuse and responded positively to intramuscular B12 supplementation. It is important to consider possible nitrous oxide abuse while investigating suspected B12 deficiency, especially in patients presenting with nonspecific myeloneuropathy. Elevated methylmalonic acid (MMA) level is specific for diagnosing B12 deficiency. Prompt diagnosis and treatment can lead to the resolution of the symptoms and prevent further nerve damage.

## Introduction

Nitrous oxide was first discovered in 1793 by Joseph Priestly, the scientist who also discovered oxygen. For over 200 years, nitrous oxide has remained one of the most widely used anesthetics in the medical field [1]. Unfortunately, it is also widely abused as an illicit recreational inhalant. It is absorbed by diffusion through the lungs and excreted via respiration. Its half-life is approximately five minutes, and it is relatively safe when used at appropriate doses under close monitoring for anesthetic effect [2]. However, several studies have shown that prolonged uncontrolled use of inhaled nitrous oxide can lead to neurotoxicity due to its adverse effect on vitamin B12 function and subsequent inhibition of methionine synthase, the enzyme responsible for generating the methyl group used in the synthesis of DNA, RNA, and myelin protein. Neurotoxicity is potentially reversible with abstinence from nitrous oxide inhalation and adequate vitamin B12 supplementation.

## Case presentation

This is a case of a 32-year-old male with a past medical history of attention deficit hyperactivity disorder, not currently on any medication, who presented to our emergency department with complaints of tingling sensation in his four extremities and leg weakness for six months, stating that “his legs gave out” while trying to get up from the couch. The patient endorsed progressively worsening paresthesia of his extremities in glove and stocking distribution, with the lower extremity involvement extending up to the abdomen at the level of the umbilicus. His symptoms were constant and aggravated by neck flexion. He endorsed chronic recreational nitrous oxide inhalation.

On examination, his vitals were stable. Neurological exam revealed intact cranial nerves, 4/5 strength in bilateral upper extremities, and 3/5 strength in bilateral lower extremities, positive Lhermitte sign, significantly diminished light touch sensation and proprioception in his extremities, absent vibratory sense in the lower extremities with grossly intact temperature and pain sensation. Also noted were normal finger-to-nose test, bilateral lower extremity hyperreflexia, and bilateral extensor plantar response. Gait was ataxic with a severe imbalance and high fall risk. Extensive laboratory studies were obtained including specific vitamin levels, autoimmune studies, thyroid function tests, diabetes screening, and Lyme serology. Results were significant for vitamin B12 324pg/mL (182-914 pg/mL), serum homocysteine 68.0 µmol/L (<11.4 µmol/L), and methylmalonic acid (MMA) 10,739 nm/L (87-318 nm/L) (Table 1).

**Table 1:** Pertinent laboratory studies obtained on admission. ANA: Antinuclear antibody

Test	Value	Reference range
White cell count	7.8 10ˆ3/µL	4.1-11.0 10ˆ3/µL
Hemoglobin	11.3 g/dL	13.5-17.5 g/dL
Hematocrit	35.4%	41.0-53.0%
Mean corpuscular volume	MCV 87.6 fL	80.0-99.0 fL
Hemoglobin A1C	5.9%	<=5.7 %
Vitamin B12	324 pg/mL	180-914 pg/mL
Folate, Serum	>20.0 ng/mL	6.0-20.0 ng/mL
Methylmalonic acid	10,739 nmol/L	87-318 nmol/L
Homocysteine	68.0 µmol/L	<11.4 µmol/L
Magnesium	2.0 mg/dL	1.7-2.8 mg/dL
Sodium	138 mmol/L	133-146 mmol/L
Potassium	4.1 mmol/L	3.5-5.1 mmol/L
Bicarbonate	28 mmol/L	21-31 mmol/L
Blood Urea Nitrogen	15 mg/dL	7-25 mg/dL
Creatinine	0.76 mg/dL	0.60-1.30 mg/dL
Calcium	9.2 mg/dL	8.4-10.2 mg/dL
Glucose, Random	113 mg/dL	70-100 mg/dL
Thyroid Stimulating Hormone	4.16 µIU/mL	0.30 - 5.33 µIU/mL
SARS-COV-2-NAT	Negative	Negative
Vitamin B1	133 nmol/L	78-185 nmol/L
Vitamin B6	22.2 ng/mL	2.1-21.7 ng/mL
Lyme antibody	<0.9 (negative)	<0.9 (negative)
ANA screen	Negative	Negative

**Figure 1:**
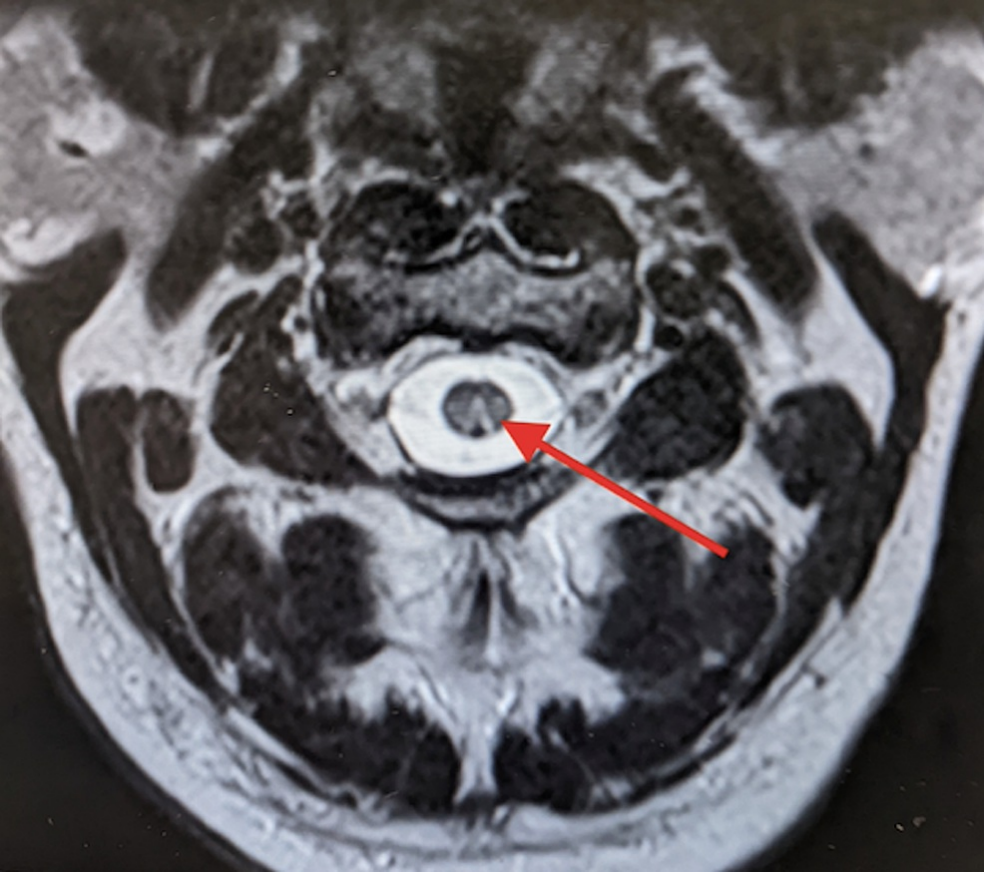
MRI axial cervical spine showing the “inverted V sign” (red arrow), consistent with SCD of the cord. MRI: Magnetic Resonance Imaging; SCD: Subacute Combined Degeneration.

Comprehensive imaging studies including magnetic resonance imaging (MRI) of the brain, cervical spine thoracic spine, and lumbar spine were obtained with findings on cervical spine MRI significant for inverted V sign, consistent with subacute combined degeneration (SCD) (Figure 1).

Electromyography (EMG) revealed moderate mixed axonal and demyelinating sensory and motor polyneuropathy. The patient was diagnosed with subacute combined spinal cord degeneration secondary to nitrous oxide-induced vitamin B12 deficiency. He was started on intramuscular cyanocobalamin 1,000 mcg/mL daily for five days with a maintenance dose of 1,000 mcg per week. He was also educated about the detrimental side effects of nitrous oxide inhalation and complete abstinence was recommended to prevent further nerve demyelination and worsening of neurological symptoms. He responded to treatment and was doing well at his one-week outpatient follow-up, during which he reported significant improvement of symptoms.

## Discussion

Vitamin B12, also called cobalamin, is an essential water-soluble vitamin that is derived from animal products such as eggs, dairy, and red meat [3]. Common causes of vitamin B12 deficiency include dietary insufficiency, malabsorption, and autoimmune pernicious anemia. Importantly, nitrous oxide can also play a role in vitamin B12 deficiency. Once absorbed, vitamin B12 is used as a coenzyme for the methylation processes in DNA, fatty acid, and myelin synthesis. The mechanism by which vitamin B12 deficiency results in demyelination is not entirely clear. Earlier studies conducted in animals, however, suggest that the primary pathophysiology is the deficiency of the methyl group due to homocysteine methyltransferase dysfunction. By oxidizing the cobalt in cobalamin, nitrous oxide inactivates vitamin B12 and consequently reduces methionine synthase activity which results in inadequate production of methionine. This causes failure of myelin maintenance with the spinal cord and axonal degeneration. Therefore, a deficiency in vitamin B12 level can lead to the development of neurologic complications including the degeneration of the dorsal and lateral white matter of the spinal cord, producing slowly progressive weakness, paresthesia, spasticity, sensory ataxia, paraplegia, and urinary incontinence in severe cases [4]. These neurologic deficits can occur without the evidence of megaloblastic anemia.

When evaluating the patient with suspected vitamin B12 deficiency or SCD, it is important to obtain a thorough history to look for maladaptive disorders such as celiac disease or Crohn's disease, surgeries including gastrectomy or ileal resection, poor dietary intake including a strict vegan diet, and social history, especially recreational nitrous oxide use. Laboratory studies including complete blood count, vitamin B12 levels, homocysteine, and MMA levels are essential to support the diagnosis. The cut-off value for total vitamin B12 level of below 200 ng/L reportedly has a 97% sensitivity for vitamin B12 deficiency, although the cut-off value can range from 135 to 473 in various reports [5]. In most cases of nitrous oxide toxicity, vitamin B12 levels are often within the normal limits. Therefore, to diagnose vitamin B12 deficiency in these individuals, measurement of MMA and homocysteine levels is more useful than total serum B12 levels. MRI is the conventional neuroimaging modality for the differential diagnosis of SCD. Most patients with SCD show no abnormality on MRI. However, as observed in our patient, signal abnormalities are usually very distinctive when present. This often appears hyperintense on T2-weighted imaging but normal on T1-weighted imaging with an unremarkable or slight enhancement [6]. Other distinctive features such as an “inverted V sign,” a “pair of binoculars sign,” and a “dot sign” have also been identified on MRI [7,8]. In addition to nitrous oxide toxicity induced SCD, other differential diagnoses of SCD may include multiple sclerosis, neuromyelitis optical, spinal tumors, copper deficiency myelopathy, and medications adverse effects from proton pump inhibitors or metformin.

Heavy recreational nitrous oxide users are at a dose-dependent risk of developing severe vitamin B12 deficiency with significant neurological complications with the most reported presentations being numbness, paresthesia, and weakness followed by ataxia, difficulty ambulating, and falls [9]. Other less frequently reported neurological sequelae caused by nitrous oxide abuse include but are not limited to mental status changes, bladder or bowel incontinence, sexual dysfunction, and a positive Lhermitte’s sign [10]. In this case, our patient endorsed chronic inhalation of nitrous oxide for at least 10 years, which could have led to progressive toxicity and the ultimate development of subacute combined spinal cord degeneration as evidenced by his clinical findings and critically elevated MMA levels. Treatment generally involves subcutaneous or intramuscular vitamin B12 replacement. Dose recommendations for severe neurological deficits include 1,000 mcg daily for one week then weekly for one month followed by maintenance therapy with 1,000 mcg monthly. Vitamin B12 and MMA levels should be monitored during therapy and the patient should be followed clinically until the resolution of symptoms.

## Conclusions

The use of recreational nitrous oxide and its potentially toxic effects is significantly under-reported in medical journals. Clinicians must make efforts to inquire about nitrous oxide abuse when investigating neuropathies, myelopathies, and suspected vitamin B12 deficiency. Prompt identification and treatment can alleviate symptoms and prevent irreversible neurological deficits.
